# Environmental variation causes different (co) evolutionary routes to the same adaptive destination across parasite populations

**DOI:** 10.1002/evl3.27

**Published:** 2017-10-17

**Authors:** Stuart K. J. R. Auld, June Brand

**Affiliations:** ^1^ Biological and Environmental Sciences University of Stirling Stirling United Kingdom

**Keywords:** Adaptation, coevolution, experimental evolution, host–parasite interactions

## Abstract

Epidemics are engines for host‐parasite coevolution, where parasite adaptation to hosts drives reciprocal adaptation in host populations. A key challenge is to understand whether parasite adaptation and any underlying evolution and coevolution is repeatable across ecologically realistic populations that experience different environmental conditions, or if each population follows a completely unique evolutionary path. We established twenty replicate pond populations comprising an identical suite of genotypes of crustacean host, *Daphnia magna*, and inoculum of their parasite, *Pasteuria ramosa*. Using a time‐shift experiment, we compared parasite infection traits before and after epidemics and linked patterns of parasite evolution with shifts in host genotype frequencies. Parasite adaptation to the sympatric suite of host genotypes came at a cost of poorer performance on foreign genotypes across populations and environments. However, this consistent pattern of parasite adaptation was driven by different types of frequency‐dependent selection that was contingent on an ecologically relevant environmental treatment (whether or not there was physical mixing of water within ponds). In unmixed ponds, large epidemics drove rapid and strong host‐parasite coevolution. In mixed ponds, epidemics were smaller and host evolution was driven mainly by the mixing treatment itself; here, host evolution and parasite evolution were clear, but coevolution was absent. Population mixing breaks an otherwise robust coevolutionary cycle. These findings advance our understanding of the repeatability of (co)evolution across noisy, ecologically realistic populations.

**Impact Summary**

Over time, populations often become better suited to their local environment as poor performing individuals are removed by natural selection. For most parasites, environment is determined mainly by the hosts they infect. As the composition of different host types change over time, so does the selection on parasite populations. Changes in host composition will also be shaped by the parasite, as hosts and parasites are locked in a coevolutionary cycle. Here, we examine the consistency of host‐parasite (co)evolution and parasite adaptation across replicate seminatural populations. Ordinarily, natural populations vary so much that it difficult to examine the repeatability of host‐parasite interactions. We used a novel approach that combines the benefits of controlled experimental manipulation with ecological realism. We established 20 freshwater crustacean populations, each of which had the same genetic composition, and exposed them to isolates of the same original population of a sterilizing bacterial parasite; half of the populations experienced a stirring treatment. At the end of the season, after all the populations suffered epidemics, we sampled each of the parasite populations and exposed them to a test set of hosts. This experiment allowed us to demonstrate that parasites adapted to host genetic types that were present in the pond populations–both in terms of ability to infect the host and within‐host parasite growth. However, the consistent pattern of adaptation masked very different dynamics that depended on the stirring treatment: in unstirred populations, parasites adapted to previously resistant hosts that became common (coevolution), whereas in stirred populations, hosts evolved, but not in terms of their resistance, and parasites adapted to hosts that were neither common nor rare (host evolution and parasite evolution). We therefore demonstrate that the relationship between adaptation and (co)evolution can vary according to environmental treatment, but is nevertheless not completely unique to individual populations.

## Introduction

Parasites commonly exert strong selection on their host populations and vice versa, driving rapid coevolutionary change (Jaenike 1978; Brockhurst et al. 2003; Koskella and Lively 2009; Paterson et al. 2010; Schulte et al. 2011; Thrall et al. 2012; Lenski and Levin 2015). These coevolutionary interactions provide a window through which to observe host and parasite adaptation to local conditions, where parasites adapt to better infect local hosts than hosts from other populations or hosts adapt to better resist local parasites relative to foreign parasites (Lively 1989; Ebert 1994; Imhoof and Schmid Hempel 1998; Kaltz and Shykoff 1998; Oppliger et al. 1999; Koskella 2014). The relative magnitude of host and parasite local adaptation depends on both the adaptive genetic variation within each population and the strength of selection from the antagonist relative to other selection pressures (Kawecki and Ebert 2004). Populations that have different histories of selection and adaptive genetic variation can exhibit similar patterns of local adaptation, and vice versa. So, while parasite local adaptation demonstrates the potential for parasite‐mediated selection on the host population (Gandon and Nuismer 2009), it cannot tell us whether that host population has sufficient genetic variation to respond to such selection. To understand the workings of the coevolutionary engine that drives local adaptation, we must examine replicate ecologically realistic populations over both space and time (Blanquart and Gandon 2013; Koskella 2014).

Patterns of host‐parasite coevolution fall along a continuum. On one extreme, arms race evolution (ARE) of increased general host resistance and parasite infectivity strips genetic variation from populations, slowing coevolution (Obbard et al. 2011) until the arrival of new genotypes as a result of mutation or immigration. On the other extreme, fluctuating selection (FS), which occurs when the likelihood of infection depends on the precise combination of host and parasite genotypes, can drive Red Queen dynamics and maintain genetic diversity in both host and parasite populations over the long term (provided the parasite is virulent; Howard and Lively 1994). Under FS, adaptation is fuelled by standing genetic variation and does not require a supply of new mutations (Hamilton 1980; Howard and Lively 1994). Theory tells us that the position of coevolutionary dynamics on the ARE‐FS spectrum is governed largely by the genetics underlying host–parasite interactions (Agrawal and Lively 2002), with implications for local adaptation. When infection depends on the precise combination of host and parasite genotypes (termed genotypic specificity), FS can emerge (Hamilton 1980) and local adaptation is strong, because increased fitness on local hosts comes at an automatic cost of reduced fitness on foreign hosts. When infection is not genotype specific, that is when parasites can infect a broad range of host genotypes, ARE is more likely and local adaptation is weaker; this is because selection for increased performance on local hosts leads to correlated selection for increased performance on other foreign hosts (Morgan et al. 2005).

Infection genetics is not the only determinant of host‐parasite coevolution. Both the nature of coevolution and its strength can depend on environmental conditions (Lazzaro and Little 2009; Wolinska and King 2009; Mostowy and Engelstädter 2011). For example, increased physical flux (mixing) within populations results in increased contact rate between bacteria and their phage parasites, accelerating coevolution (Brockhurst et al. 2003) and selecting on the phage to infect a broader range of host genotypes; this causes shifts coevolutionary dynamics from FS to ARE (Gómez et al. 2015). Environmental variation among populations means the mode and tempo of coevolution can potentially vary between ARE to FS, or break down into host evolution and/or parasite evolution occurring in isolation (Blanford et al. 2003; Mostowy and Engelstädter 2011; Harrison et al. 2013), leading to different patterns of local adaptation (Laine 2007, 2008). If coevolution shifts from FS to ARE, one expects increases in host range would mean parasites perform better on both local and foreign hosts, reducing the strength of local adaptation. However, to adequately test this theory, we require a better understanding of how (co)evolution and adaptation are linked across replicate populations in more ecologically complex, that is more natural, settings (Laine 2007; Thrall et al. 2012; Koskella 2014; Bankers et al. 2017; Gibson et al. 2017).

We established twenty replicate outdoor pond populations of the crustacean host, *Daphnia magna*, and its natural bacterial parasite, *Pasteuria ramosa*. In this system, infection depends on genotypic specificity (Luijckx et al. 2011), so there is the potential for FS dynamics and parasite local adaptation to emerge in these populations (Decaestecker et al. 2007). Each pond was seeded with the same suite of host genotypes and isolates from the same genetically diverse parasite population. Ponds experienced natural environmental variation over space and time, and half‐experienced a physical flux (population mixing) treatment (known to reduce infection prevalence in this system; Auld and Brand 2017) to extend the test for mixing‐mediated shifts in (co)evolution beyond phage‐bacteria systems. We then dissected the relationship between parasite adaptation and host evolution, parasite evolution, and host‐parasite coevolution using a time‐shift experiment (Gaba and Ebert 2009), where a test set of host genotypes were exposed to ancestral parasite isolates and to isolates collected from the pond populations at the end of the epidemic (similar to Auld et al. 2014a). By combining laboratory experimental data on changes in infection traits with outdoor experimental data on shifts in host genotype frequencies, we dissected host evolution of resistance, parasite evolution of infectivity, and within‐host growth and the overall change in parasitism due to coevolution over the course of the epidemic. We found consistent parasite local adaptation—in terms of ability to infect and grow within the host—across populations. This adaptation was underpinned by strong coevolution in unmixed ponds, but separate host evolution and parasite evolution in mixed ponds.

## Methods

### STUDY ORGANISMS

*Pasteuria ramosa* is a Gram positive bacterial endoparasite. *Pasteuria* transmission spores are ingested by filter‐feeding *Daphnia magna*, and cause infection when they bind to and penetrate the host gut epithelium (Duneau et al. 2011; Auld et al. 2012a). Once inside the host, the spores grow and sporulate (Auld et al. 2014bc), causing host sterilization (Cressler et al. 2014). The parasite is an obligate killer, and millions of transmission spores are then released into the environment upon the death of the infected host (Ebert et al. 1996). *Daphnia magna* is a cyclically parthenogenetic freshwater crustacean that inhabits shallow ponds and lakes across Europe and commonly suffers infection with *Pasteuria*. Infection is easy to diagnose: *Pasteuria‐*infected *Daphnia* have obvious red‐brown bacterial growth in their haemolymph, lack developed ovaries or offspring in their brood chamber and often exhibit gigantism.

### OUTDOOR POND EXPERIMENT

We established twenty 1000 liter artificial ponds in August 2014 and allowed them to naturally fill with rainwater over an eight‐month period (Auld and Brand 2017). On the 2nd April 2015, we seeded each pond with an identical suite of 12 unique *Daphnia* genotypes (determined using microsatellite genotyping; see Auld and Brand 2017). There were ten *Daphnia* per genotype (total = 120 *Daphnia* per pond) and a genetically diverse inoculum of 1 × 10^8^ *Pasteuria* spores. This *Pasteuria* starting population was generated by exposing sediment samples to 21 genotypes of local *Daphnia*, harvesting the infected hosts, and re‐exposing transmission spores to healthy hosts for multiple rounds of infection (Auld and Brand 2017). After a two‐week establishment period, we estimated the density of *Daphnia* life stages (juveniles, healthy adults, *Pasteuria‐*infected adults) in each pond on a weekly basis. We did this by passing a 0.048 m^2^ pond net across the diameter of the mesocosm (1.51 m) and counting the resulting *Daphnia*. All *Daphnia* were returned to the ponds after counting. Half of the ponds experienced a weekly population mixing (physical flux) treatment, where mixed ponds were stirred once across the middle and once around the circumference with a 0.35 m^2^ paddle submerged halfway into the pond (the exception to this was on day one of the experiment, when all ponds experienced the mixing treatment to ensure hosts and parasites were distributed throughout the ponds).

At the end of the season, after disease epidemics had peaked in all of the populations (November 17th, 2015) we sampled 90 infected *Daphnia* from each pond population; these samples were individually homogenized and the resulting spore solutions were pooled into three isolates per pond (i.e., where each isolate consisted of 30 homogenized infected hosts), and frozen at –20°C for use in the laboratory experiment. We also sampled 20–30 *Daphnia* from 16 of the 20 ponds (10 unmixed and six mixed) for population genetic analysis (low population densities prevented us from sampling all 20 ponds). These hosts were stored individually in 70% EtOH and later genotyped at 15 microsatellite loci (Auld and Brand 2017).

### LABORATORY EXPERIMENT

We maintained replicates of a test set of fifteen *Daphnia* host genotypes with which we examined changes in *Pasteuria* infectivity and within‐host growth with respect to the corresponding ancestral isolate. First, we established maternal lines for these host genotypes. Twelve of the genotypes were the same as those hosts used to establish the pond populations (named 11A, 12A, 4A, 5B, 6A, 7A, 8A, 9A, K2B, K3A, M1B, and M2A) and three genotypes were not present in the ponds but were from the same natural host population (named K1A, M3A, and M4A). There were three replicates per genotype; each replicate consisted of eight adult *Daphnia* in 100 mL of artificial media (Klüttgen et al. 1994). Hosts were maintained in a state of clonal reproduction for three generations to minimize variation due to maternal effects, and were fed 0.5 ABS chemostat‐grown *Chlorella vulgaris* algae per *Daphnia* per day (ABS refers to the optical absorbance of 650 nm white light by the *C. vulgaris* culture). Jars were incubated at 20°C on a 12L:12D light cycle, and their media was changed three times per week. Second clutch neonates formed the experimental replicates.

The experimental design consisted of a factorial manipulation of these hosts and parasites. We crossed the 15 host genotypes with parasite isolates collected from each of the 20 ponds at the end of the outdoor experiment, plus isolates of the ancestral parasite used to inoculate the ponds at the beginning of the season. On the day of treatment exposure neonates from each maternal line were allocated to parasite treatments following a split‐clutch design. There were three replicate parasite isolates per host genotype and thus a total of 945 experimental jars (7560 *Daphnia*). Each jar received a dose of 2 × 10^5^ *Pasteuria* spores and kept under identical conditions as the maternal lines. After 48 hours exposure to the *Pasteuria* spores, the experimental *Daphnia* were transferred into fresh media. The infection status of each *Daphnia* was determined by eye 25 days post exposure, and infected *Daphnia* were then stored at –20°C. Counts of *Pasteuria* transmission spores were later determined with a haemocytometer.

### ANALYSIS

Both the outdoor experiment data (host genotype frequency, epidemic size) and laboratory experiment data (parasite infectivity, within‐host growth) were analyzed using the R statistical package (R Core Team 2013). First, we tested for rapid parasite adaptation over the course of the season, where parasites perform better on host genotypes with which they share a recent coevolutionary history. We did this by calculating the change in infectivity and spore burdens between the ancestral parasites and the parasite samples collected at the end of the season and then fitting linear‐mixed effects models (LMMs) to these data; host type (sympatric/allopatric), and population flux treatment (mixed/unmixed) were fitted as fixed effects, and the pond population‐by‐host genotype interactions were fitted as random effects with separate intercepts for each host type. Then we fitted a LMM to test whether the change in parasite spore burden was associated with the change in infectivity; once again, pond population‐by‐host genotype interactions were fitted as random effects with separate intercepts for each host type.

Next, we examined how parasite adaptation covaried with host genotype frequencies at the end of the season (for the 16 ponds for which we had data on host genotype frequencies). Once again, we fitted LMMs to the data for changes in parasite traits, but this time we included final host genotype frequency (along with second‐order polynomial), physical flux treatment (mixed/unmixed) and their interaction as fixed effects; here, the pond population‐by‐host genotype interactions were fitted as random effects with separate intercepts for each population flux treatment. For all LMMs, we applied a Satterthwaite approximation to account for different variances across treatment groups.

By combining data on change in parasite infectivity and spore burden with multilocus genotype frequency data, we dissected the effects of epidemic on: (1) how host populations evolved resistance to the ancestral parasite population (in terms of infectivity and spore burden); (2) how the parasite evolved infectivity and the capacity to proliferate within infected hosts of the ancestral host population; and (3) how coevolution shaped the proportion of infected hosts and spore burdens within evolved host and parasite populations. First, we estimated the relative fitness of host genotypes in each pond population by determining their relative frequency (eq. (1)):

(1) $${\bar{w}}_{h,\;t}=P_{h,t}.n_{h},$$

where *P_h,t_* refers to the frequency of host genotype *h* at time *t*, and *n_h_* is the total number of host genotypes used to seed the population (in this case, *n_h_* =12). So, at the beginning of the epidemic (*t =* 0), all host genotypes have a relative fitness of 1. At the end, the relative fitness of each host genotype varies within and across populations.

For each pond population, we calculated the mean change in host susceptibility to infection (Δ*i_h_*), and change in the parasite burden in infected hosts (Δ*s_h_*) that were exposed to the ancestral parasite (eq. (2a), (2b)):

(2a) $$\Delta i_{\;h}=\frac{1}{n}.\sum_{h}\left(\left(i_{h,t=0}.{\bar{w}}_{h,t=1}\right)-i_{h,t=0}\right),$$

(2b) $$\Delta s_{h}=\frac{1}{n}.\sum_{h}\left(\left(s_{h,t=0}.{\bar{w}}_{h,t=1}\right)-s_{h,t=0}\right),$$

where *i_h,t_* is the proportion of hosts *h* in each pond that suffer infection at time *t* (Note that the parasite isolates are identical across populations when *t =* 0.) *n* is the number of pond populations and *s_h,t_* refers to the spore burden on infected hosts *h* in each pond. Then, we calculated the change in parasite infectivity (Δ*i_p_*), and within host spore burdens (Δ*s_p_*) for parasite populations that were exposed to the ancestral host population (eq. (3a), (3b)):

(3a) $$\Delta i_{\;p}=\frac{1}{n}.\sum_{h}\left(i_{h,t=1}-i_{h,t=0}\right),$$

(3b) $$\Delta s_{h}=\frac{1}{n}.\sum_{h}\left(s_{h,t=1}-s_{h,t=0}\right),$$

Next, we calculated the population‐level change in infectivity and spore burden that would result from host‐parasite coevolution, by weighting the change in parasite traits by the change in host genotype frequencies (eq. (4a), (4b)).

(4a) $$\Delta i_{\;hp}=\frac{1}{n}.\sum_{h}\left(\left(i_{h,t=1}.{\bar{w}}_{h,t=1}\right)-i_{h,t=0}\right),$$

(4b) $$\Delta s_{hp}=\frac{1}{n}.\sum_{h}\left(\left(s_{h,t=1}.{\bar{w}}_{h,t=1}\right)-s_{h,t=0}\right).$$

Finally, we dissected the population‐level effects of epidemic size on host evolution of susceptibility to infection and parasite within‐host growth (Δ*i_h_*, Δ*s_h_*), parasite evolution of infectivity and within‐host growth (Δ*i_p_*, Δ*s_p_*), and the coevolutionary outcomes in terms of likelihood of infection and parasite burdens (Δ*i_hp_*, Δ*s_hp_*). We did this by fitting linear models (LMs) to each of the six response variables with epidemic size included as a fixed effect.

## Results

### PARASITE ADAPTATION TO SYMPATRIC HOST POPULATIONS

**Figure 1:**
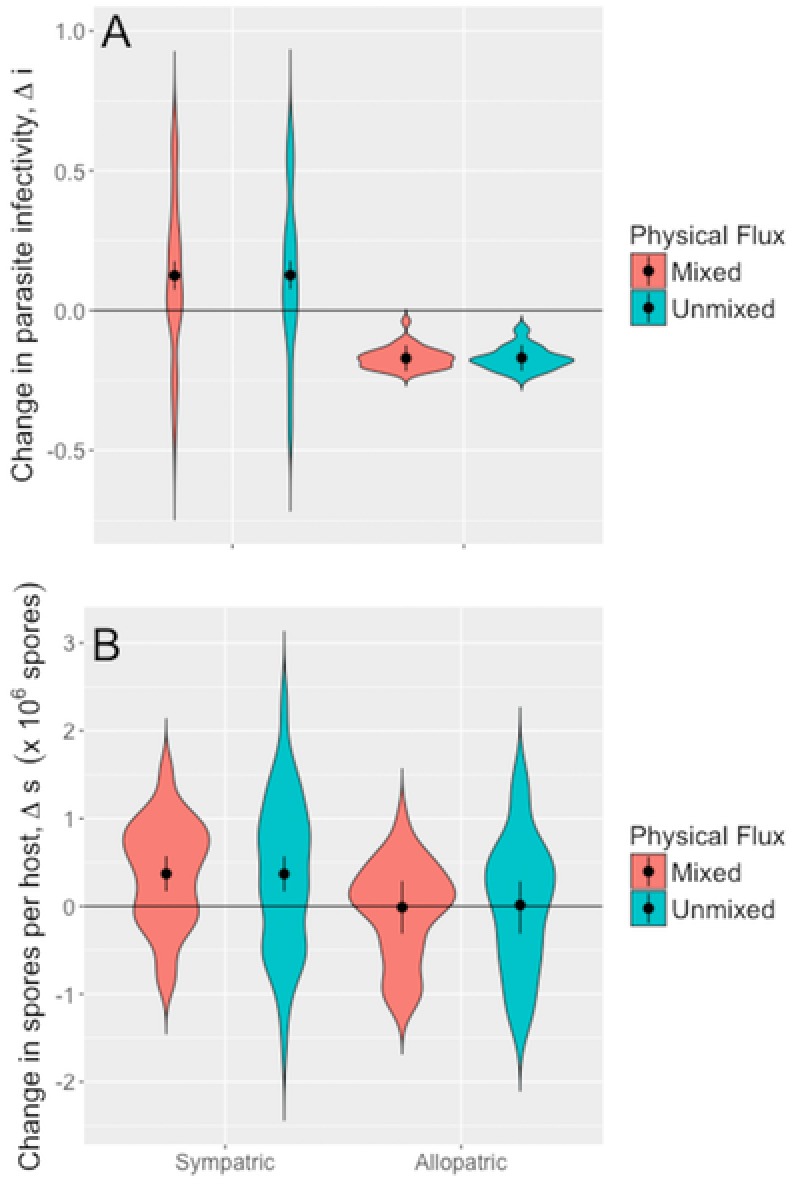
Change in (A) parasite infectivity; and (B) within‐host growth in parasites taken from mixed (*n =* 10) and unmixed (*n =* 10) populations when exposed to sympatric hosts (genotypes that were present in the pond populations) and allopatric hosts (related genotypes that were not present in the pond populations). Violin plots show the distribution of the raw data; points and bars denote the means and 95% confidence intervals predicted by the LMM.

After just a single epidemic, a striking pattern of rapid parasite adaption emerged across populations. Findings from our laboratory experiment revealed parasite isolates from the end of the season evolved to be more infectious than the ancestral parasite population when exposed to the host genotypes present in the pond populations (sympatric hosts), but significantly less infectious when exposed to novel host genotypes with which they had not interacted with (allopatric hosts) (linear‐mixed effects model, LMM: *F*_1, 228.94_ = 110.92, *P* < 0.0001; Fig. 1A; Fig. S1A; Table S1). These changes in parasite infectivity did not differ according to physical flux treatment (LMM: *F*_1, 158.8_ = 0.003, *P* = 0.96; Fig. 1A; Fig. S1A; Table S1).

There was also an increase in the number of parasite spores per infected host in sympatric hosts over the season, but no change in spore burdens in allopatric hosts (LMM: *F*_1, 103.32_ = 6.24, *P* < 0.014; Fig. 1B; Fig. S1B; Table S2); again, this did not differ across physical flux treatments (LMM: *F*_1, 295.68_ = 0.003, *P* = 0.99; Fig. 1B; Fig. S1B; Table S2). The variance explained by the parasite population by host genotype interaction was higher for parasites exposed to sympatric hosts (0.89) than for parasites exposed to allopatric hosts (0.55). Finally, we found a strong positive relationship between the change in spore burden and change in infectivity (Fig. S2, LMM: *F*_1,550.58_ = 221.39, *P* < 0.0001).

### PARASITE EVOLUTION AND HOST GENOTYPE FREQUENCIES

**Figure 2:**
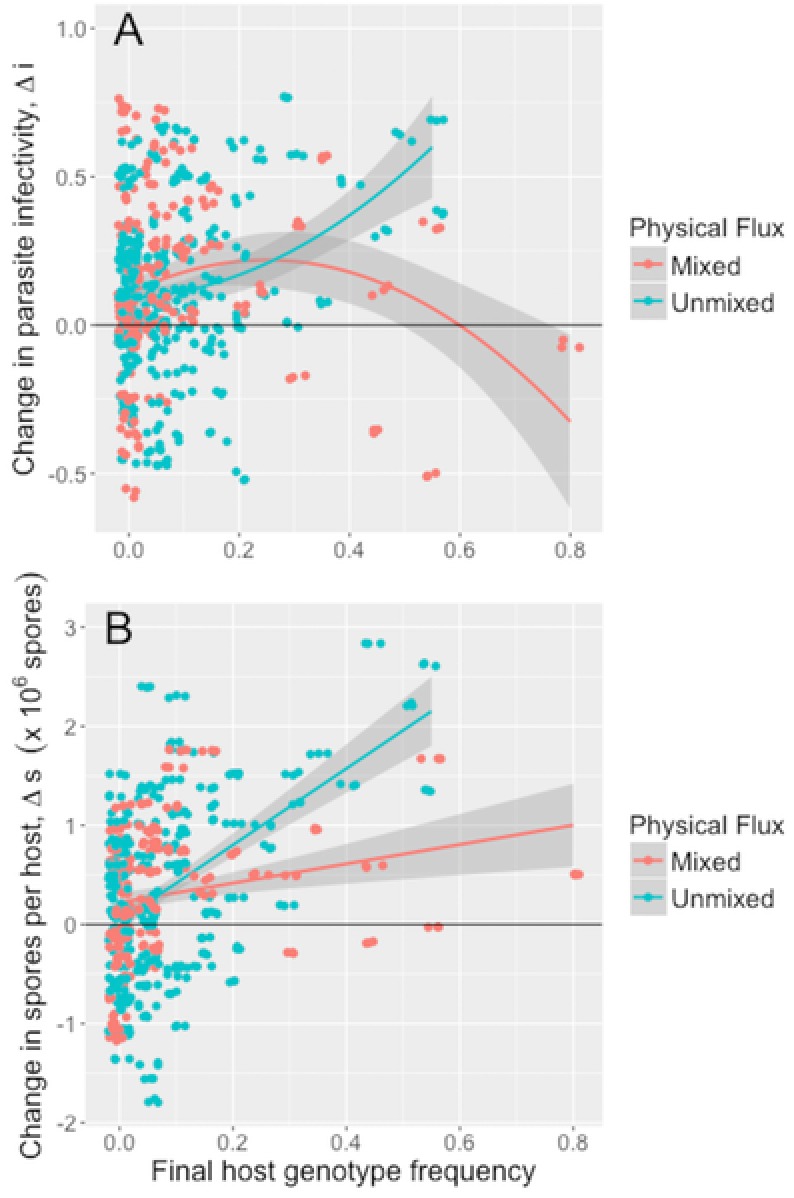
Associations between change in infection traits and host genotype frequencies in sympatric host populations at the end of the epidemic. (A) change in parasite infectivity, and (B) change in within‐host growth (between parasite isolates collected after epidemics and corresponding ancestral isolates) taken from mixed (*n* = 6) and unmixed (*n* = 10) populations. Lines and shaded bands denote the slopes and 95% confidence intervals predicted by the LMM; points are jittered for clarity.

Despite near‐identical local adaption across physical flux treatments, we uncovered different underlying patterns of host‐parasite (co)evolution. After pairing data from both the outdoor and laboratory experiment, we uncovered a strong positive relationship between the change in parasite infectivity over the season and final host genotype frequency in unmixed ponds; in mixed populations, increases in parasite infectivity were highest on host genotypes at intermediate final frequencies (quadratic effect of host genotype frequency × physical flux interaction; LMM: *F*_1, 163.91_ = 4.19, *P* = 0.017; Fig. 2A; Table S3). The proportion of the variance in change in infectivity explained by the parasite population by host genotype interaction was similar in unmixed (39%) and mixed populations (44%). We found a strong positive relationship between the change in the number of parasite spores per infected host over the season and final host genotype frequency in unmixed ponds, and a very weak positive relationship in mixed ponds (host genotype frequency × physical flux interaction; LMM: *F*_1, 189.25_ = 5.25, *P* = 0.023; Fig. 2B; Table S4). The proportion of the variance in change in within‐host parasite burden explained by the parasite population by host genotype interaction was higher in unmixed (32%) than in mixed populations (23%).

### DISSECTING HOST EVOLUTION, PARASITE EVOLUTION, AND COEVOLUTION

**Figure 3:**
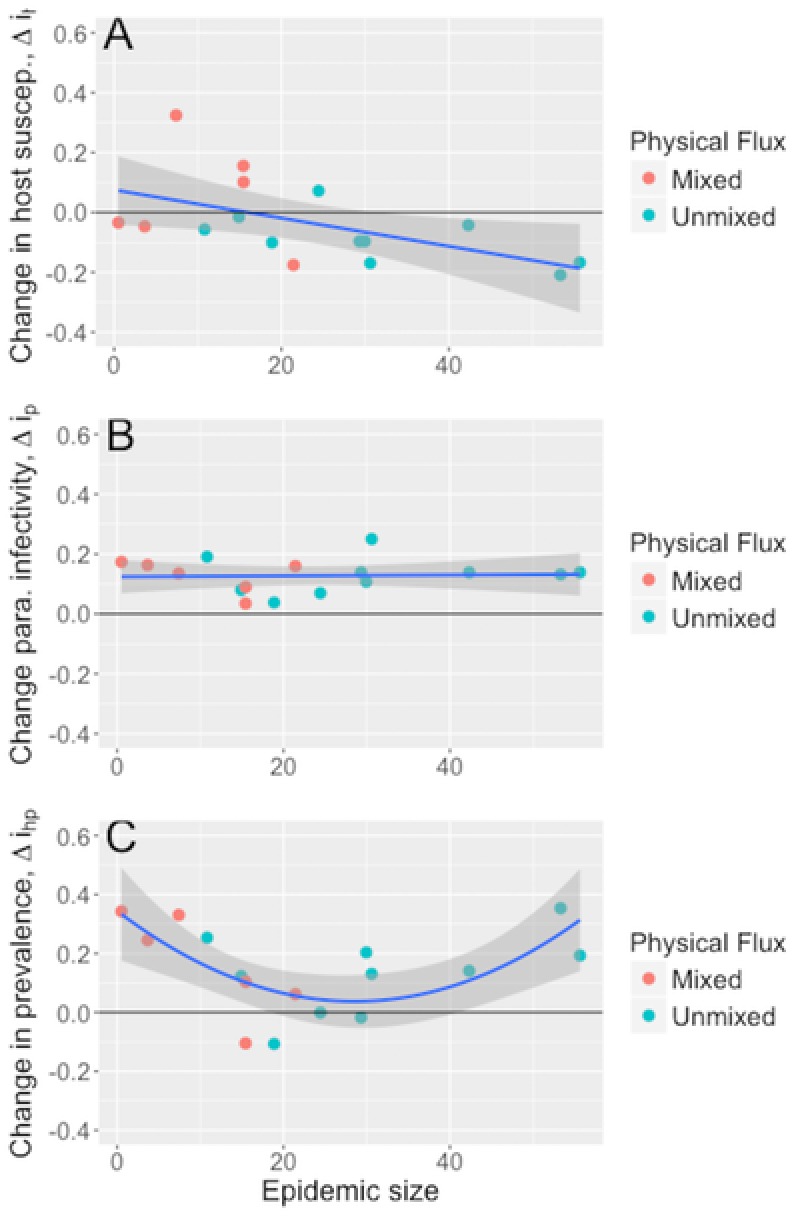
Effect of epidemic size on population‐level coevolution of infectivity (*n* = 16). (A) Host susceptibility (susceptibility to the ancestral parasite weighted by relative shifts in host genotype frequencies), (B) parasite infectivity (mean infectivity of postepidemic parasite isolates assuming fixed host genotype frequencies), and (C) coevolution of overall infection risk (mean infectivity of postepidemic parasite isolates weighted by relative shifts in host genotype frequencies). Lines and shaded bands denote the predicted slopes and 95% confidence intervals predicted by each LM.

By combining data on infectivity and spore burden in both ancestral and evolved parasite populations with multilocus genotype frequency data, we dissected the population‐level effects of epidemic size on: (1) how host populations evolved resistance to the ancestral parasite population; (2) how the parasite evolved infectivity and the capacity to proliferate within infected hosts of the ancestral host population (here host genotype frequencies are fixed); and (3) how coevolution shaped the proportion of infected hosts and spore burdens within evolved host and parasite populations. We found larger epidemics were associated with the evolution of reduced host susceptibility to infection (i.e. increased resistance) with the ancestral parasite (Δ*i_h_*, linear model, LM: *F*_1,14_ = 5.97, *P* = 0.028; Fig. 3A). The evolution of increased parasite infectivity over the season was not associated with epidemic size (Δ*i_p_*, LM: *F*_1,14_ = 0.02, *P* = 0.88; Fig. 3B). The overall change in infection risk (i.e., the change in parasite infectivity when weighted by the final host genotype frequency) exhibited a quadratic relationship with epidemic size: ponds that experienced either small or large epidemics showed an increase in infection risk, whereas ponds that experienced epidemics of an intermediate size showed no overall change in infection risk (Δ*i_hp_*, LM: *F*_2,13_ = 6.40, *P* = 0.012; Fig. 3C).

**Figure 4:**
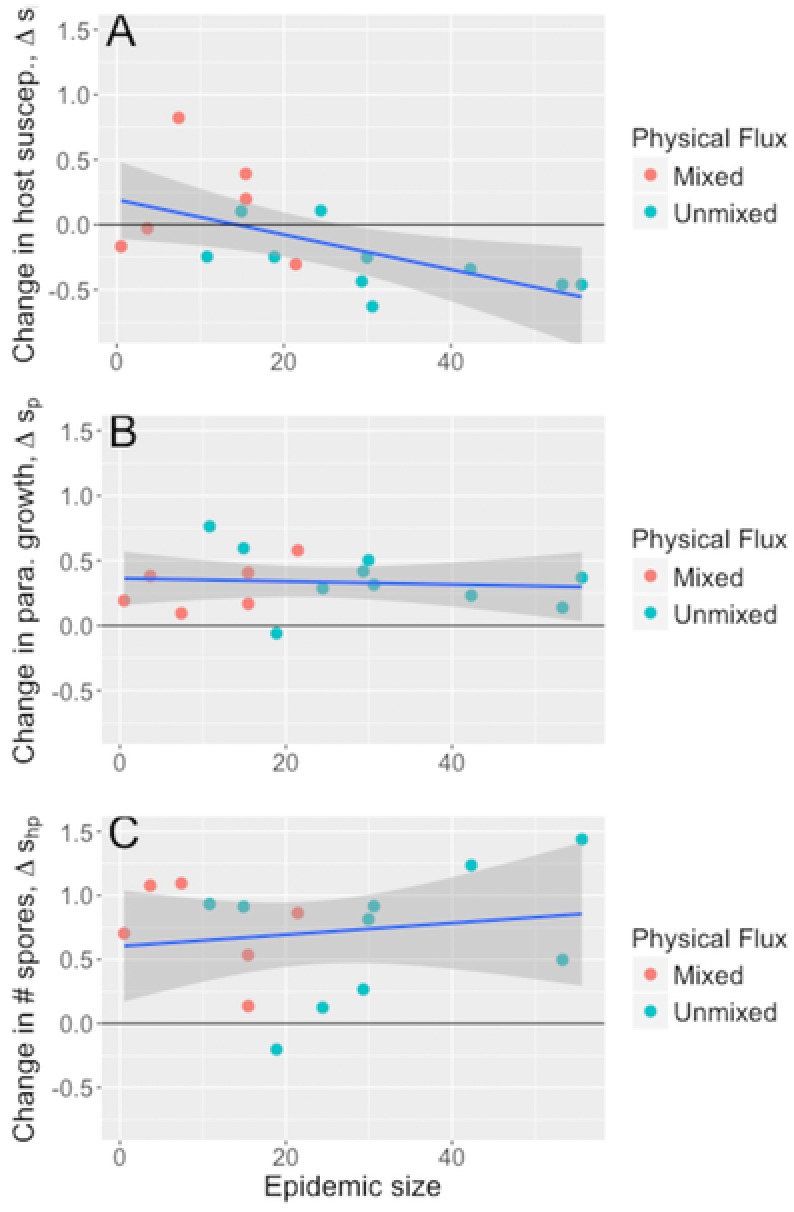
Effect of epidemic size on population‐level coevolution of parasite within‐host growth (*n* = 16). (A) Host susceptibility (mean spore burdens from the ancestral parasite weighted by relative shifts in host genotype frequencies), (B) parasite within‐host growth (mean spore burdens from postepidemic parasite isolates assuming fixed host genotype frequencies), and (C) coevolution of overall parasite burden (mean spore burdens from postepidemic parasite isolates weighted by relative shifts in host genotype frequencies). Lines and shaded bands denote the predicted slopes and 95% confidence intervals predicted by each LM.

Epidemic size had a subtly different effect on the coevolutionary patterns of parasite within‐host growth and host resistance to it. Similar to the infectivity data, larger epidemics were associated with the evolution of reduced spore burdens in hosts infected with the ancestral parasite (Δ*s_h_* , LM: *F*_1,14_ = 7.31, *P =* 0.017; Fig. 4A). The evolution of increased parasite within‐host growth over the season was not associated with epidemic size (Δ*s_p_*, LM: *F*_1,14_ = 0.12, *P* = 0.74; Fig. 4B). Finally, there was an increase in the overall change in spore burden (i.e., the change in spores per infected host when weighted by the final host genotype frequency) over the season, but this was not associated with epidemic size (Δ*s_hp_*, LM: *F*_1,14_ = 0.387, *P* = 0.54; Fig. 4C).

## Discussion

Natural selection commonly drives populations to become adapted to their local environment. Parasites are no different, although there is the added complication that their principal environment, the host, is also evolving in response to selection (Kawecki and Ebert 2004; Schulte et al. 2011). Moreover, in many host‐parasite systems, evolution and adaptation occurs in rapid bursts during disease epidemics (Duffy et al. 2009; Penczykowski et al. 2016). We examined the changes in parasite infection traits across replicate semi‐natural populations of *Daphnia magna* and their castrating parasite *Pasteuria ramosa*, and tested if any signatures of adaptation and (co)evolution were either consistent over space, dependent on physical flux, or specific to each individual population. We identified consistent signatures of parasite local adaptation that emerged after just a single epidemic. However, this adaptation was driven by coevolution in unmixed ponds and parasite evolution only in mixed ponds.

*Pasteuria* populations evolved to better infect sympatric host genotypes at a cost of being poorer at infecting allopatric host genotypes (i.e., hosts which they have not shared a recent coevolutionary history) over the epidemic (Fig. 1A). The emergence of rapid local adaptation and foreign maladaptation is expected, given the known infection genetics in *Daphnia‐Pasteuria* systems: alleles that allow a parasite to infect one set of host genotypes lead to an inability to infect other host genotypes (Carius et al. 2001; Auld et al. 2012b, but see Luijckx et al. 2011). The infection genetics in this system is therefore consistent with the matching allele (MA) model of infection (Grosberg and Hart 2000; Bento et al. 2017) and is known to exhibit FS dynamics in the long term (Decaestecker et al. 2007).

The parasite burden data revealed a different pattern: there was evidence of parasite local adaptation to sympatric hosts, but this did not come at a cost of maladaptation to allopatric hosts (Fig. 1B). Previous work has shown that within‐host parasite growth varies across parasite genotypes, but does not exhibit genotypic specificity (Vale and Little 2009). So, while infectivity is governed by MA genetics, within‐host parasite growth is likely to be a quantitative trait that depends mainly on parasite genotype and its interactions with other environmental conditions (though change in infectivity and change in within‐host growth are correlated; Fig. S2). Whether or not infection occurs is by far the most important step in the parasite transmission process, because infected hosts are sterilized and failure of the parasite to bind to the host gut leads to a failure of parasite replication. Therefore, the most intense fluctuating selection occurs for resistant/infectivity alleles at this initial step; alleles for parasite replication will experience less host‐mediated selection, because any variation in fitness among infected hosts is minimal when compared to variation between infected and healthy hosts (Ebert et al. 2004).

We expected the physical flux treatment to increase contact rate between hosts and parasites (May and Anderson 1979) leading to larger epidemics, stronger parasite‐mediated selection and greater parasite adaptation (Morgan et al. 2005), as found in a phage‐bacteria system (Gómez et al. 2015). However, adaptation was equally strong across physical flux treatments even though epidemics were smaller in mixed ponds than in unmixed ponds (Fig. 1A). This is because the host populations in mixed ponds experience direct selection from the mixing treatment (Auld and Brand 2017), with downstream consequences for parasite evolution and adaptation. An examination of coevolutionary patterns across 16 of the 20 populations (i.e., populations for which there was host genotype frequency data) revealed that physical flux changed the nature of host‐mediated selection on the parasite population. Previous work has found that coevolutionary paths are idiosyncratic to individual populations (Schulte et al. 2011), whereas we find that coevolution is consistent across the unmixed populations, and that parasite evolution and host evolution is consistent across the mixed populations. Host genotypes either increase or decrease in frequency in a generally consistent manner across populations of each mixing treatment, and measures of drift are comparatively low (Auld and Brand 2017). Also, in unmixed populations, the parasite evolved increased infectivity and within‐host growth when exposed to host genotypes that became more common, but not when exposed to hosts that became rare (Fig. 2A, B). By contrast, in mixed populations, the parasite best adapted to infect host genotypes that achieved intermediate frequency and grew best within hosts that became rare (Fig. 2A, B).

Although the (co)evolutionary paths differ according to physical flux treatment, we find evidence that FS on parasite infectivity is operating across the board: the proportion of random effects variance explained by a host genotype by population interaction is consistent across physical flux treatments, which tells us there is no overall shift in host range (as would be predicted by ARE models: Betts et al. 2014). The same is not true for the change in parasite burden: the variance explained by the host genotype by population interaction is much lower in mixed ponds than in unmixed ponds, indicating the parasite is more of a generalist in mixed ponds in terms of within‐host growth (Gómez et al. 2015). Both ARE and FS models of coevolution predict that parasite populations should adapt to common host genotypes after a lag, and that parasites should generally perform best on hosts from the recent past (Jaenike 1978; Hutson and Law 1981; Nee 1989; Sasaki 2000; Lively 2016). Empirical data from invertebrate‐trematode (Dybdahl and Lively 1998; Koskella and Lively 2009), bacteria‐phage (Koskella 2014) and *Daphnia‐Pasteuria* (Decaestecker et al. 2007) systems support these theoretical predictions; our findings further demonstrate that this adaptation can occur extremely rapidly, within a single epidemic.

By weighting the changes in parasite infectivity and within‐host growth by shifts in host genotype frequency, we were able to dissect host evolution, parasite evolution and host‐parasite coevolution. Our previous study demonstrated that unmixed populations suffered larger epidemics than mixed populations (Auld and Brand 2017). Here, we found larger epidemics (in unmixed ponds) selected for host resistance to the ancestral parasite, both in terms of infectivity and within‐host growth (Fig. 3A, Fig. 4A), consistent with previous studies (Duncan et al. 2006; Duffy et al. 2012). By contrast, epidemic size had no effect on the mean change in parasite infectivity of within‐host growth (Fig. 3B, Fig. 4B). The change in overall infection risk as a result of host‐parasite coevolution revealed how coevolution varied across epidemics of differing size, and that parasite‐mediated selection and host‐mediated selection were often not equal in magnitude. In mixed populations that suffered small epidemics, hosts remained susceptible while parasites evolved increased infectivity and growth, leading to higher overall infection risk (Fig. 3A); host evolution did not result in adaptation to the ancestral parasite, whereas parasite evolution did result in adaptation to the local suite of host genotypes. In unmixed populations, infection risk increased with epidemic size: large epidemics and strong parasite‐mediated selection for host resistance was outweighed by parasite evolution of increased infectivity. Moreover, the parasite evolved to be proportionally better at infecting host genotypes that were resistant to the ancestral parasite, demonstrating coevolution (Fig. 2A).

Host and parasite adaptation depends on the supply of adaptive genetic variation and the strength of antagonist‐mediated selection relative to other selective forces. Host‐mediated selection is equally strong across populations irrespective of epidemic size and environmental conditions, whereas parasite‐mediated selection was much stronger in unmixed populations because they experienced larger epidemics. The host generally provides the principal environment and thus the main selective force acting on parasite infectivity and growth, whereas host populations experience a wide range of different selective forces in addition to selection for resistance to parasitism. Unmixed ponds, with their large epidemics and symmetric parasite‐ and host‐mediated selection, were “coevolutionary hotspots.” By contrast, mixed ponds with their smaller epidemics, weak parasite‐mediated selection and strong host‐mediated selection were “coevolutionary coldspots” (Thompson 2005). Nevertheless, these environment‐dependent signatures of evolution and coevolution result in similar patterns of adaptation across parasite populations, demonstrating a certain level of repeatability across noisy environments.

Associate Editor: R. Snook

## Supporting information


**Table S1**. Linear mixed effects model analysing effects of physical flux and host type on the change in parasite infectivity over the course of an epidemic.
**Table S2**. Linear mixed effects model analysing effects of physical flux and host type on the change in parasite within‐host growth over the course of an epidemic.
**Table S3**. Linear mixed effects model analysing effects of physical flux and final host genotype frequency on the change in parasite within‐host growth over the course of an epidemic.
**Table S4**. Linear mixed effects model analysing effects of physical flux and final host genotype frequency on the change in parasite within‐host growth over the course of an epidemic.
**Figure S1**. Consequences of exposure of 15 host genotypes exposed to 21 parasite samples. (A) proportion of hosts infected; and (B) within‐host parasite growth within infected hosts (millions of spores). Twelve of 15 host genotypes were sympatric (present in the pond populations) and three were allopatric (not present in the pond populations). Twenty of the parasite populations were sampled from the ponds at the end of the epidemic (10 from mixed and 10 from unmixed), and one consisted of the ancestral parasite population used to seed the ponds. There were three replicate isolates per parasite population.
**Figure S2**. Relationship between the change in parasite within‐host growth (spore burden) and change in parasite infectivity (as predicted by the LMM).

Supporting Information

Supporting Information
