# Supplementary material for: Environmental variation causes different (co) evolutionary routes to the same adaptive destination across parasite populations
Source: Evol Lett. 2017 Oct 17;1(5):245–54. doi: 10.1002/evl3.27 (PMC6121849; doi:10.1002/evl3.27)
Supplement: Supplementary file 1 — Table S1. Linear mixed effects model analysing effects of physical flux and host type on the change in parasite infectivity over the course of an epidemic. Table S2. Linear mixed effects model analysing effects of physical flux and host type on the change in parasite within‐host growth over the course of an epidemic. Table S3. Linear mixed effects model analysing effects of physical flux and final host genotype frequency on the change in parasite within‐host growth over the course of an epidemic. Table S4. Linear mixed effects model analysing effects of physical flux and final host genotype frequency on the change in parasite within‐host growth over the course of an epidemic. Figure S1. Consequences of exposure of 15 host genotypes exposed to 21 parasite samples. (A) proportion of hosts infected; and (B) within‐host parasite growth within infected hosts (millions of spores). Twelve of 15 host genotypes were sympatric (present in the pond populations) and three were allopatric (not present in the pond populations). Twenty of the parasite populations were sampled from the ponds at the end of the epidemic (10 from mixed and 10 from unmixed), and one consisted of the ancestral parasite population used to seed the ponds. There were three replicate isolates per parasite population. Figure S2. Relationship between the change in parasite within‐host growth (spore burden) and change in parasite infectivity (as predicted by the LMM). [file EVL3-1-245-s001.docx]

*Supporting information*

| Table S1. Linear mixed effects model analysing effects of physical flux and host type on the change in parasite infectivity over the course of an epidemic. |
| --- |
| \| Change in infectivity \| df \| *χ^2^* \| *P* \| \| --- \| --- \| --- \| --- \| \| Fixed effects \|  \|  \|  \| \| Physical flux (Unmixed/Mixed) \| 1 \| 0.0025 \| 0.96 \| \| Host type (Sympatric/Allopatric) \| 1 \| 110.9254 \| <0.0001 \| \| Flux x Host \| 1 \| 0.0138 \| 0.91 \| \| Random effects \| Variance \| SD \|  \| \| Pond x genotype (Sympatric) \| 0.095 \| 0.308 \|  \| \| Pond x genotype (Allopatric) \| 0.006 \| 0.075 \|  \| |

| Table S2. Linear mixed effects model analysing effects of physical flux and host type on the change in parasite within-host growth over the course of an epidemic. |
| --- |
| \| Change in within-host growth \| df \| *χ^2^* \| *P* \| \| --- \| --- \| --- \| --- \| \| Fixed effects \|  \|  \|  \| \| Physical flux (Unmixed/Mixed) \| 1 \| 0.0002 \| 0.99 \| \| Host type (Sympatric/Allopatric) \| 1 \| 6.2419 \| 0.012 \| \| Flux x Host \| 1 \| 0.0996 \| 0.75 \| \| Random effects \| Variance \| SD \|  \| \| Pond x genotype (Sympatric) \| 0.887 \| 0.942 \|  \| \| Pond x genotype (Allopatric) \| 0.546 \| 0.739 \|  \| |

| Table S3. Linear mixed effects model analysing effects of physical flux and final host genotype frequency on the change in parasite within-host growth over the course of an epidemic. |
| --- |
| \| Change in infectivity \| df \| *χ^2^* \| *P* \| \| --- \| --- \| --- \| --- \| \| Fixed effects \|  \|  \|  \| \| Final host genotype freq.2 \| 2 \| 5.1025 \| 0.08 \| \| Physical flux (Unmixed/Mixed) \| 1 \| 0.0509 \| 0.82 \| \| Flux x Final host freq2 \| 2 \| 8.3781 \| 0.0152 \| \| Random effects \| Variance \| SD \|  \| \| Pond x genotype (Unmixed) \| 0.087 \| 0.295 \|  \| \| Pond x genotype (Mixed) \| 0.095 \| 0.307 \|  \| |

| Table S4. Linear mixed effects model analysing effects of physical flux and final host genotype frequency on the change in parasite within-host growth over the course of an epidemic. |
| --- |
| \| Change in within-host growth \| df \| *χ^2^* \| *P* \| \| --- \| --- \| --- \| --- \| \| Fixed effects \|  \|  \|  \| \| Final host genotype freq \| 1 \| 12.7288 \| <0.001 \| \| Physical flux (Unmixed/Mixed) \| 1 \| 0.0906 \| 0.76 \| \| Flux x Final host freq2 \| 1 \| 5.2542 \| 0.022 \| \| Random effects \| Variance \| SD \|  \| \| Pond x genotype (Unmixed) \| 0.974 \| 0.987 \|  \| \| Pond x genotype (Mixed) \| 0.711 \| 0.843 \|  \| |

|  |
| --- |
| Figure S1. Consequences of exposure of 15 host genotypes exposed to 21 parasite samples. (A) proportion of hosts infected; and (B) within-host parasite growth within infected hosts (millions of spores). Twelve of 15 host genotypes were sympatric (present in the pond populations) and three were allopatric (not present in the pond populations). Twenty of the parasite populations were sampled from the ponds at the end of the epidemic (10 from mixed and 10 from unmixed), and one consisted of the ancestral parasite population used to seed the ponds. There were three replicate isolates per parasite population. |

|  |
| --- |
| Figure S2. Relationship between the change in parasite within-host growth (spore burden) and change in parasite infectivity (as predicted by the LMM). |
