# Supplementary material for: Environmental variation causes different (co) evolutionary routes to the same adaptive destination across parasite populations
Source: Evol Lett. 2017 Oct 17;1(5):245–54. doi: 10.1002/evl3.27 (PMC6121849; doi:10.1002/evl3.27)
Supplement: Supplementary file 2 — Supporting Information [file EVL3-1-245-s002.pdf]

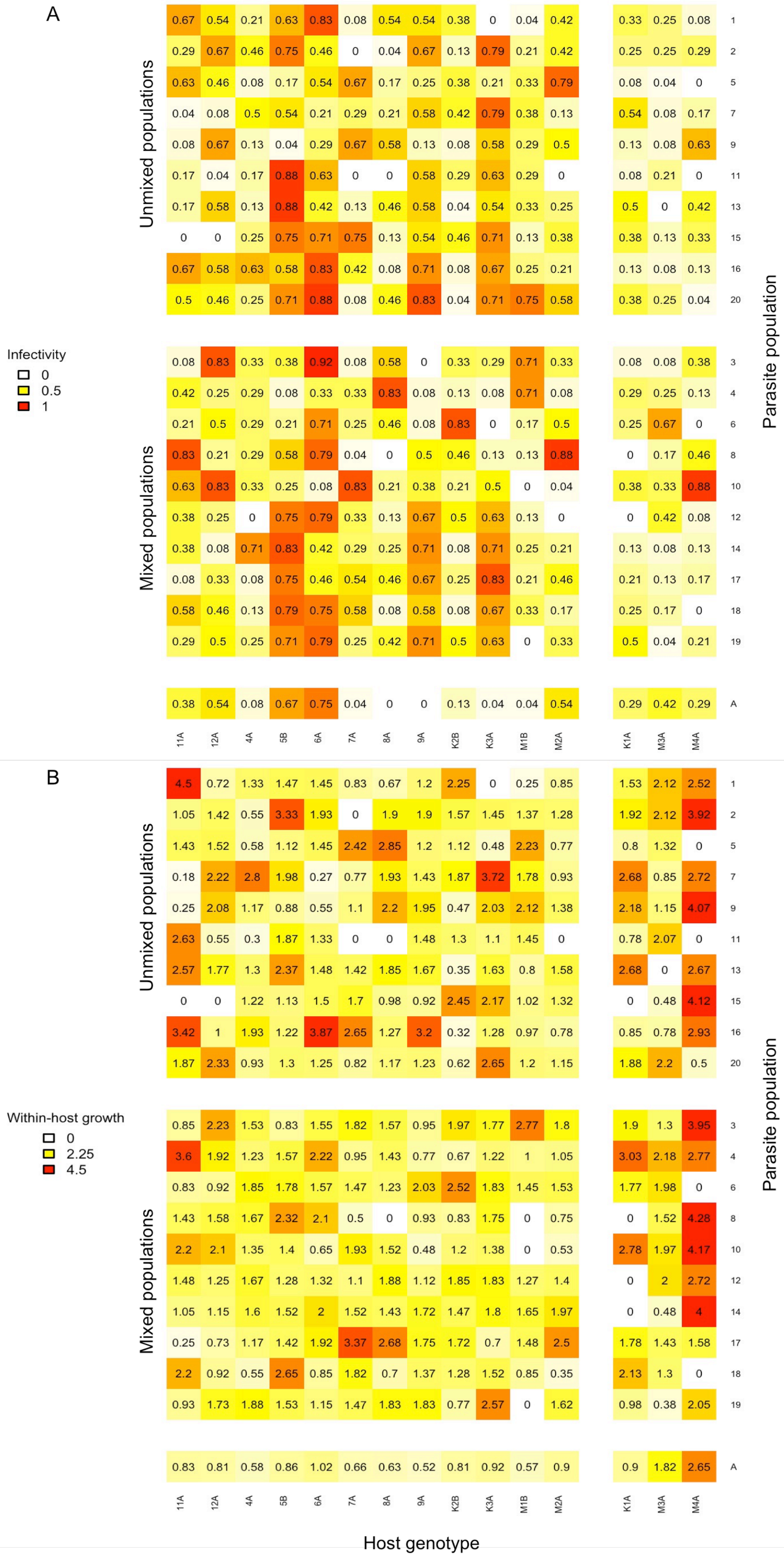
A
Unmixed populations
Mixed populations
Parasite population
Infectivity
0
0.5
1
0.67 0.54 0.21 0.63 0.83 0.08 0.54 0.54 0.38 0 0.04 0.42 0.33 0.25 0.08 1
0.29 0.67 0.46 0.75 0.46 0 0.04 0.67 0.13 0.79 0.21 0.42 0.25 0.25 0.29 2
0.63 0.46 0.08 0.17 0.54 0.67 0.17 0.25 0.38 0.21 0.33 0.79 0.08 0.04 0 5
0.04 0.08 0.5 0.54 0.21 0.29 0.21 0.58 0.42 0.79 0.38 0.13 0.54 0.08 0.17 7
0.08 0.67 0.13 0.04 0.29 0.67 0.58 0.13 0.08 0.58 0.29 0.5 0.13 0.08 0.63 9
0.17 0.04 0.17 0.88 0.63 0 0 0.58 0.29 0.63 0.29 0 0.08 0.21 0 11
0.17 0.58 0.13 0.88 0.42 0.13 0.46 0.58 0.04 0.54 0.33 0.25 0.5 0 0.42 13
0 0 0.25 0.75 0.71 0.75 0.13 0.54 0.46 0.71 0.13 0.38 0.38 0.13 0.33 15
0.67 0.58 0.63 0.58 0.83 0.42 0.08 0.71 0.08 0.67 0.25 0.21 0.13 0.08 0.13 16
0.5 0.46 0.25 0.71 0.88 0.08 0.46 0.83 0.04 0.71 0.75 0.58 0.38 0.25 0.04 20
0.08 0.83 0.33 0.38 0.92 0.08 0.58 0 0.33 0.29 0.71 0.33 0.08 0.08 0.38 3
0.42 0.25 0.29 0.08 0.33 0.33 0.83 0.08 0.13 0.08 0.71 0.08 0.29 0.25 0.13 4
0.21 0.5 0.29 0.21 0.71 0.25 0.46 0.08 0.83 0 0.17 0.5 0.25 0.67 0 6
0.83 0.21 0.29 0.58 0.79 0.04 0 0.5 0.46 0.13 0.13 0.88 0 0.17 0.46 8
0.63 0.83 0.33 0.25 0.08 0.83 0.21 0.38 0.21 0.5 0 0.04 0.38 0.33 0.88 10
0.38 0.25 0 0.75 0.79 0.33 0.13 0.67 0.5 0.63 0.13 0 0 0.42 0.08 12
0.38 0.08 0.71 0.83 0.42 0.29 0.25 0.71 0.08 0.71 0.25 0.21 0.13 0.08 0.13 14
0.08 0.33 0.08 0.75 0.46 0.54 0.46 0.67 0.25 0.83 0.21 0.46 0.21 0.13 0.17 17
0.58 0.46 0.13 0.79 0.75 0.58 0.08 0.58 0.08 0.67 0.33 0.17 0.25 0.17 0 18
0.29 0.5 0.25 0.71 0.79 0.25 0.42 0.71 0.5 0.63 0 0.33 0.5 0.04 0.21 19
0.38 0.54 0.08 0.67 0.75 0.04 0 0 0.13 0.04 0.04 0.54 0.29 0.42 0.29 A
11A 12A 4A 5B 6A 7A 8A 9A K2B K3A M1B M2A K1A M3A M4A
B
Unmixed populations
Mixed populations
Parasite population
Within-host growth
0
2.25
4.5
4.5 0.72 1.33 1.47 1.45 0.83 0.67 1.2 2.25 0 0.25 0.85 1.53 2.12 2.52 1
1.05 1.42 0.55 3.33 1.93 0 1.9 1.9 1.57 1.45 1.37 1.28 1.92 2.12 3.92 2
1.43 1.52 0.58 1.12 1.45 2.42 2.85 1.2 1.12 0.48 2.23 0.77 0.8 1.32 0 5
0.18 2.22 2.8 1.98 0.27 0.77 1.93 1.43 1.87 3.72 1.78 0.93 2.68 0.85 2.72 7
0.25 2.08 1.17 0.88 0.55 1.1 2.2 1.95 0.47 2.03 2.12 1.38 2.18 1.15 4.07 9
2.63 0.55 0.3 1.87 1.33 0 0 1.48 1.3 1.1 1.45 0 0.78 2.07 0 11
2.57 1.77 1.3 2.37 1.48 1.42 1.85 1.67 0.35 1.63 0.8 1.58 2.68 0 2.67 13
0 0 1.22 1.13 1.5 1.7 0.98 0.92 2.45 2.17 1.02 1.32 0 0.48 4.12 15
3.42 1 1.93 1.22 3.87 2.65 1.27 3.2 0.32 1.28 0.97 0.78 0.85 0.78 2.93 16
1.87 2.33 0.93 1.3 1.25 0.82 1.17 1.23 0.62 2.65 1.2 1.15 1.88 2.2 0.5 20
0.85 2.23 1.53 0.83 1.55 1.82 1.57 0.95 1.97 1.77 2.77 1.8 1.9 1.3 3.95 3
3.6 1.92 1.23 1.57 2.22 0.95 1.43 0.77 0.67 1.22 1 1.05 3.03 2.18 2.77 4
0.83 0.92 1.85 1.78 1.57 1.47 1.23 2.03 2.52 1.83 1.45 1.53 1.77 1.98 0 6
1.43 1.58 1.67 2.32 2.1 0.5 0 0.93 0.83 1.75 0 0.75 0 1.52 4.28 8
2.2 2.1 1.35 1.4 0.65 1.93 1.52 0.48 1.2 1.38 0 0.53 2.78 1.97 4.17 10
1.48 1.25 1.67 1.28 1.32 1.1 1.88 1.12 1.85 1.83 1.27 1.4 0 2 2.72 12
1.05 1.15 1.6 1.52 2 1.52 1.43 1.72 1.47 1.8 1.65 1.97 0 0.48 4 14
0.25 0.73 1.17 1.42 1.92 3.37 2.68 1.75 1.72 0.7 1.48 2.5 1.78 1.43 1.58 17
2.2 0.92 0.55 2.65 0.85 1.82 0.7 1.37 1.28 1.52 0.85 0.35 2.13 1.3 0 18
0.93 1.73 1.88 1.53 1.15 1.47 1.83 1.83 0.77 2.57 0 1.62 0.98 0.38 2.05 19
0.83 0.81 0.58 0.86 1.02 0.66 0.63 0.52 0.81 0.92 0.57 0.9 0.9 1.82 2.65 A
11A 12A 4A 5B 6A 7A 8A 9A K2B K3A M1B M2A K1A M3A M4A
Host genotype
