# Supplementary figures and images for: Environmental variation causes different (co) evolutionary routes to the same adaptive destination across parasite populations
Source: Evol Lett. 2017 Oct 17;1(5):245–54. doi: 10.1002/evl3.27 (PMC6121849; doi:10.1002/evl3.27)

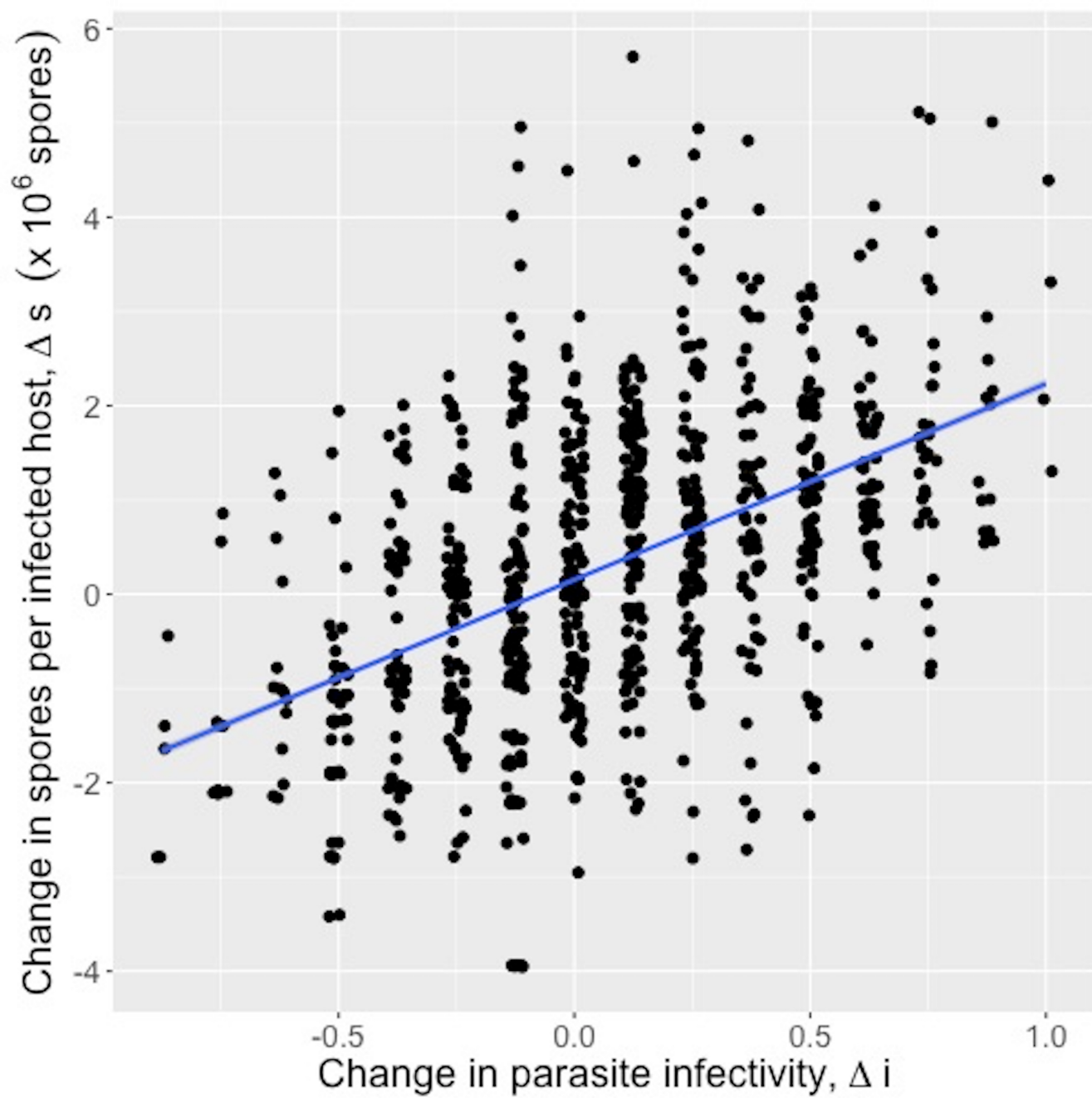

Supplement: Supplementary file 3 — Supporting Information [file EVL3-1-245-s003.pdf]
